# Multivariate analysis of management and biosecurity practices in smallholder pig farms in Madagascar

**DOI:** 10.1016/j.prevetmed.2009.08.010

**Published:** 2009-11-15

**Authors:** S. Costard, V. Porphyre, S. Messad, S. Rakotondrahanta, H. Vidon, F. Roger, D.U. Pfeiffer

**Affiliations:** a Epidemiology Division, Department of Veterinary Clinical Sciences, Royal Veterinary College, Hawkshead Lane, North Mymms, Hatfield, Hertfordshire AL9 7TA, UK; b Centre de Coopération Internationale en Recherche Agronomique pour le Développement (CIRAD), Campus International de Baillarguet, 34398 Montpellier Cedex 5, France; c Malagasy des Professionnels de l’Elevage (MPE), BP 579, Antananarivo, Madagascar; d International Livestock Research Institute, P.O. Box 30709-00100 Nairobi, Kenya

**Keywords:** Multivariate analysis, Multiple factor analysis, Hierarchical cluster analysis, Management practices, Biosecurity

## Abstract

A cross-sectional study was carried out in 2005 and 2006 in three geographical areas of Madagascar to investigate and differentiate swine farm management and biosecurity practices in smallholder farming communities. Questionnaire data from a total of 709 pig farms were analysed using multiple factor analysis (MFA) and hierarchical cluster analysis (HCA). Variables describing management and biosecurity practices were organised into five groups: structure of the farm, animal-contacts, person- and vehicle-contacts, feeding, and sanitary aspects. In general, few biosecurity measures were implemented in the pig farms included in the study. Regional differences in management and biosecurity practices emerged from the MFA and were mainly due to, in order of decreasing importance: structure of the farm, sanitary aspects, feeding and animal-contacts and, to a lesser extent, person- and vehicle-contacts. HCA resulted in the differentiation of four distinct types of farms in each of two study areas, Arivonimamo and Marovoay, while no grouping could be identified amongst farms in Ambatondrazaka area. The characterisation of the different types of smallholder pig farms will allow adapting recommendations on husbandry practices and control measures in pig farms of these regions of Madagascar. The development of tailored recommendations is essential for Malagasy smallholders who have limited resources and need to make evidence-based management changes to reduce the risk of contagious diseases in their herds.

## 1 Introduction

In Madagascar, pig production is very important for smallholder farmers, for subsistence as well as commercial purposes. The pig production sector suffered severe losses following the introduction of African swine fever (ASF) in the late 1990s (Rousset et al., 2001). Currently, its re-establishment is adversely affected by regular outbreaks of contagious diseases, such as ASF, classical swine fever (CSF) and Teschen disease (Serres and Ramisse, 1969; Randriamparany et al., 2005).

In order to reduce the risk of disease in their swine herds and consequently improve their livelihoods, Malagasy smallholders need to increase on-farm biosecurity – protection from the introduction of infectious agents (Amass and Clark, 1999). ASF, CSF and Teschen disease are transmitted by direct and indirect contacts between pigs, swill feeding, fomites, and *Ornithodoros* ticks in the case of ASF. The risk for these diseases to be introduced into a herd is therefore likely to be influenced by multiple aspects of management and biosecurity practices such as pig sales and purchases, feeding regimes, and visitors allowed onto premises. A description of the management and biosecurity practices currently used in Malagasy pig farms is an important prerequisite to the investigation of their association with disease risk. Furthermore, the identification and characterisation of different profiles of management and biosecurity practices will allow the development of tailored recommendations for pig farmers to reduce the risk of disease in their herds.

Biosecurity and management practices are measured by a large number of variables, many of which are correlated. Multivariate exploratory analyses have been used to investigate these practices in swine farms (Hurnik et al., 1994; Rose and Madec, 2002; Boklund et al., 2004; Ribbens et al., 2008) because of their ability to extract key information from large datasets and understand correlation between variables. The current study uses multiple factor analysis (MFA) (Escofier and Pages, 1994) together with cluster analysis. MFA analyses several groups of variables defined for the same set of observations and expresses the relationships existing between the groups of variables. In this study, variables were grouped according to distinct aspects of husbandry practices, aspects which were assumed to have a similar influence on the potential risk of introduction of contagious disease on the farm. MFA resulted in the identification of the main aspects of management and biosecurity practices differentiating pig farms. Cluster analysis was subsequently used to identify groups of farms with similar practices and describe their characteristics.

Using a questionnaire survey, the objectives of this study were:

• To describe management and biosecurity practices of Malagasy smallholder pig farms and investigate whether important differences existed between geographical areas.
• To investigate whether distinct types of pig farms could be differentiated on the basis of these practices, and to characterise these.

## 2 Materials and methods

### 2.1 Study sample

**Fig. 1:**
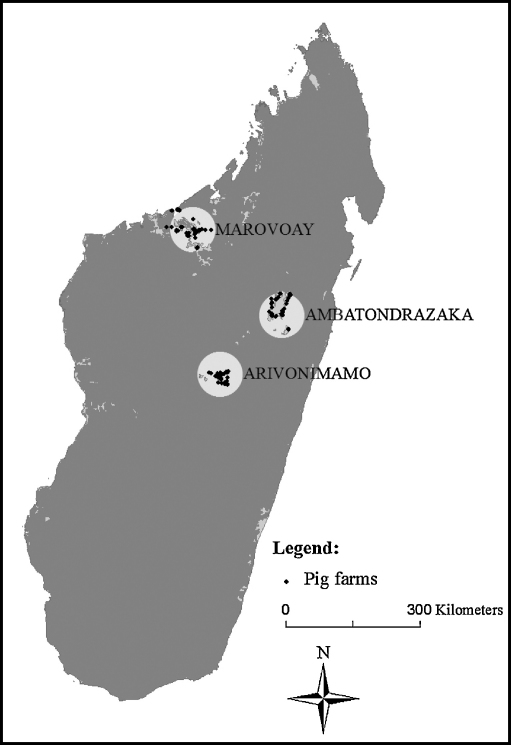
Study areas selected in Madagascar for the description of swine farm management and biosecurity practices: Marovoay, Ambatondrazaka and Arivonimamo. Points represent the 709 pigs farms included in the multivariate analysis.

A cross-sectional study was conducted in Madagascar from December 2005 to April 2006 in three geographical areas. These areas were selected purposively based on the following criteria. First, the study areas were stratified according to the three main climatic zones of Madagascar (Table 1). Then, they are important regions for pig production where outbreaks of swine diseases are regularly reported by pig farmers. Finally, the selected areas were relatively easy to access, in order to limit logistic constraints for the data collection. As shown in Fig. 1, the study areas were named after their largest towns: Ambatondrazaka, Arivonimamo and Marovoay.

The unit of interest in this study was the individual pig farm, defined as any premise where at least one pig was reared. Our target population comprised of all such pig farms in the three study areas. A sample size of 300 pig farms per study area was specified as a target which would allow estimating husbandry practice frequencies at a 95% confidence level with a precision of 5%, assuming frequencies of 50% for dichotomous factors. Within each study area, since no register of pig farms was available, pig farms were selected using a multi-stage sampling approach. First, as primary sampling units a purposive sample of fokontanies – the smallest administrative unit in Madagascar – was selected. In each study area, the sample of fokontanies covered rural and urban areas, with settlements connected by commercial exchanges. Because of the limited road network, fokontanies with few human settlements or which were in remote locations were excluded. The secondary sampling units were the pig farms within the selected fokontanies of the three study areas. The number of pig farms to select in each fokontany was calculated assuming random proportional sampling, based on a constant sampling fraction for each study area. For this purpose, the total number of pig farms in each selected fokontany had been estimated with the assistance of veterinarians, local associations supporting pig farmers and local administrators. The sampling fraction was 20% in Ambatondrazaka, 30% in Arivonimamo and 17.5% in Marovoay. Since no sampling frame was available for the secondary sampling units, the selection of pig farms in each fokontany was done along line transects. Each line transect started in the middle of the fokontany, its direction was chosen randomly, and all pig farms of the fokontany situated along the transect were selected. The same approach was repeated until the sample size was reached for each fokontany.

### 2.2 Data collection

A questionnaire was developed to collect data on husbandry practices. Using 49 closed and semi-closed questions, the following aspects of pig farms were investigated: demographics, housing, commercial exchanges, method(s) of reproduction, contacts with other animals, contacts with people and vehicles, feeding, animal health care, waste handling and biosecurity measures.

The questionnaire was developed in French, and administered in Malagasy to ensure that farmers would understand all the questions (the questionnaire in French is available upon request to the first author). It was pre-tested with 10 pig farmers and 3 interviewers outside our study areas and questions were refined according to feedback from both farmers and interviewers. The final questionnaire was administered by 9 interviewers – 3 per study area – trained before the start of the study. For biosecurity reasons, interviewers did not enter the premises and the collected information relied solely on farmers’ responses. It was emphasised that the answers would be processed anonymously, and that correctness of the answers was necessary for the study results to be sufficiently meaningful so that they are suitable for informing the development of tailored recommendations for farmers.

### 2.3 Data analysis

MFA (Escofier and Pages, 1994) examines the relationships existing between variables separated into different groups. It can be considered as a factor analysis (principal component analysis for quantitative variables, multiple correspondence analysis for qualitative variables) applied to the whole set of variables within which each group is weighted. All elements of the dataset (individuals, variables, groups of variables) are represented graphically in a Euclidean space. The principle of factor analysis is to define projections – or factors – representing an optimised quantitative summary of the relationships between variable categories. A factor is therefore a linear combination of the variables and is characterised by its eigenvalue, which indicates the variance – or inertia – of the data it represents. The first factor is the projection which represents the highest amount of variance, and each other factor is defined so that it captures the variance not explained by the previous factor. The cumulative percentage of inertia of a given number of factors indicates the percentage of variance of the dataset they explain. Factor analysis can include both active variables, used to calculate factors, and supplementary variables, not used to define factors but projected on these factors. In a MFA, factor analyses are first performed independently for each group of variables. The results are then normalised by dividing individual scores by the square root of the first eigenvalue, in order to make the different groups of variables comparable in a global analysis. A factor analysis is then performed for the merged dataset (obtained by juxtaposing the individual normalised datasets), where each group of variables has an equal *a priori* influence. By generating common factors for both variables and groups of variables, MFA takes into account the heterogeneity of groups of variables in terms of biological meaning, and allows the identification of the main variables and groups of variables that differentiate between the individuals.

Data entry and data coding were performed using Epi Info 3.3.2, data manipulation using Microsoft Access 2003, and descriptive statistics using STATA 9.2 (Statistical Software: Release 9.2., Stata Corp., College Station, TX, USA). Multivariate analyses were conducted with the statistical software R 2.5.1 (R Development Core Team, 2007), using the package ade4 for MFA (Chessel et al., 2004; Dray et al., 2007).

A total of 42 variables describing farm management and biosecurity practices were included in the dataset. They were grouped according to different aspects of husbandry practices assumed to have the same influence on the risk of introduction of contagious diseases: 5 variables described the structure of the farm, 8 referred to contacts with other animals and pigs from other farms, 7 considered person- and vehicle-contacts, 7 referred to feeds, and 7 focused on sanitary aspects. Eight variables illustrating the demographics of the farm were also included. Binary variables for which less than 5% of farmers gave a positive answer were excluded from the analysis. Otherwise, these variables with few observations may be outliers and have a dominant influence on the definition of factors. Farms with missing values were also excluded from the analysis, as factor analyses such as MFA do not allow missing values.

MFA was conducted on data from all pig farms as well as separately for each study area, in order to identify the main aspects of management and biosecurity practices discriminating farms and investigate whether these differed between study areas. The number of factors selected for interpretation was determined using the scree plot, which indicates the eigenvalue of each consecutive factor. A break on the scree plot separates factors with large and small eigenvalues. Only factors with large eigenvalues were retained for interpretation. Hierarchical cluster analysis (HCA) (Everitt, 1974) was then used to differentiate groups of farms with similar management and biosecurity practices. HCA were conducted on pig farms’ MFA scores, using Ward's criteria for linkage. The principle of Ward's aggregation method for the HCA is to group individuals – pig farms – in a way that minimises intra-cluster variance and maximise inter-cluster variance. The characterisation of the farming practices significantly associated with each group of farms was then done by calculating test-values (Morineau, 1984). Test-values measure the distance between the within-group value and the overall value for each variable category – or practice.

## 3 Results

### 3.1 Descriptive statistics

Data on management and biosecurity practices were obtained from a total of 853 pig farms, 709 of which had no missing values and were included in the analysis: 272 farms from Ambatondrazaka (38.3%), 233 from Arivonimamo (32.9%) and 204 from Marovoay (28.8%).

The 42 categorical variables describing management and biosecurity practices were separated into 5 active groups and a group of supplementary variables. Tables 2–6 list the 34 variables separated into the following groups: structure of the farm, animal-contacts, person- and vehicle-contacts, feeding, and sanitary aspects. The 8 variables introduced as supplementary variables are presented in Table 7. Six variables were excluded from the analysis: use of proper farm clothing (0.6%), use of disinfection baths or sprays at the entrance of the premises (3.3%), quarantine for pigs entering the farm (3.6%), presence of a sickbay (0.9%), equipment shared with other farm (s) (4.0%), systematic working route progressing from clean to dirty zones of the farm (0.7%).

### 3.2 Multiple factor analysis

**Fig. 2:**
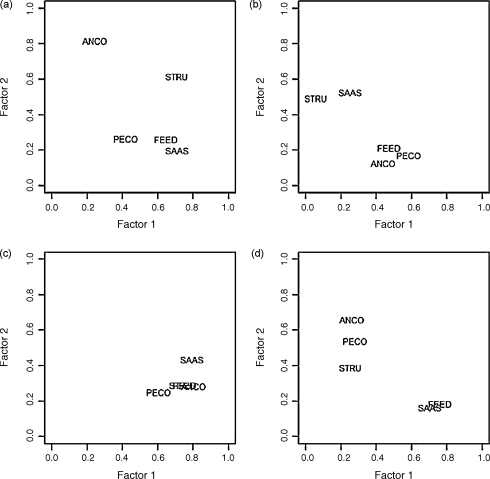
Global display of the 5 groups of variables on the two first factors of MFAs for: (a) all observations, (b) Ambatondrazaka, (c) Arivonimamo, (d) Marovay. For each group of variables, their coordinates (between 0 and 1) indicate the percentage of inertia explained by the first factor (horizontally) and the second factor (vertically). STRU: structure of the farm; ANCO: animal-contacts; PECO: person- and vehicle-contacts; FEED: feeding; SAAS: sanitary aspects.

The global displays of groups (Fig. 2) present the groups of variables in a two-dimensional space defined by the 2 first factors for each of the MFAs performed. The global display indicates the importance of the groups of variables for differentiating between pig farms: the larger their inertia on the factors 1 and 2, the more they differentiate between pig farms. For the MFA conducted on the 709 farms, the cumulative percentage of inertia for the 2 first factors was 19.2%. The husbandry practices differentiating between farms were, in decreasing order of importance: structure of the farm, sanitary aspects, feeding, animal-contacts and, to a lesser extent, person- and vehicle-contacts (Fig. 2a). For MFAs performed separately on data from Ambatondrazaka, Arivonimamo and Marovoay, we selected factors explaining 22.5, 36.5 and 19.3% of the variation, respectively. For Ambatondrazaka, Fig. 2b showed that the inertia of the five groups of variables in relation to factors 1 and 2 were low, and therefore pig farms were poorly differentiated. The plot for Arivonimamo (Fig. 2c) indicated that pig farms were differentiated by the five groups of variables on factor 1. In Marovoay, aspects of husbandry and biosecurity practices discriminating farms were, in decreasing order of importance: feeding, sanitary aspects, animal-contacts and, to a lesser extent, person-contacts (Fig. 2d). The results of the MFAs performed on data from the 3 different study areas thus showed that the management and biosecurity practices discriminating between pig farms differed greatly between regions.

**Fig. 3:**
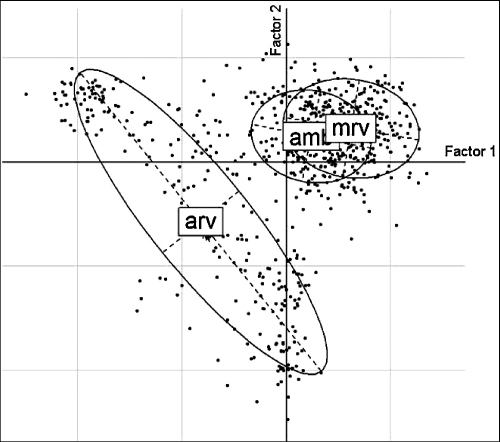
Representation of pig farms and study areas (amb: Ambatondrazaka, arv: Arivonimamo, mrv: Marovoay) in the two-dimensional space defined by the first two factors of the MFA performed on all pig farms (*n* = 709). Points represent pig farms and the distance between them is an indication of their similarity in terms of husbandry practices.

In Fig. 3, the representation of all pig farms and study areas in relation to the first two factors of the MFA showed that groups of farms with similar practices existed, and this grouping seemed related to the study areas.

The results from the MFAs therefore suggested that the main husbandry practices differed between regions.

### 3.3 Hierarchical cluster analysis

The HCAs carried out on each study area's MFA scores resulted in the identification of four clusters of farms in each of two study areas (Arivonimamo and Marovoay), while no clustering of farms was found in Ambatondrazaka. Although this latter area was not homogeneous in terms of husbandry practices, no clear groupings of farms could be identified. The main characteristics defining the clusters of farms are presented in Table 8, and a description of the clusters is provided below.

#### 3.3.1 Cluster 1: small farms with numerous animal-contacts in Arivonimamo (*n* = 81)

This was the largest cluster of farms in the region of Arivonimamo. The majority of farms were small finishing units (71.6%) with pigs of local breeds (92.6%), where less than 10 pigs were sold in 2005 (95.1%). Animals were either kept in the basement of their owner's house (53.1%), or in small pens with mud walls and thatched roof (42.0%). In most farms, replacement animals were bought in live animal markets or from other farmers (87.7%), and cattle were present on the premises (91.4%). On these farms, pigs were fed with domestic waste (93.8%) and crop by-products (88.9%), and manure was collected to fertilise crops (96.3%). In 93.8% of these farms, people were involved in other activities related to the pig production sector.

#### 3.3.2 Cluster 2: small farms with reduced health care in Arivonimamo (*n* = 42)

These were usually finishing farms (61.2%) or breeding units (23.8%) where animals of local breeds were kept in pens with mud floor (92.9%) and mud walls (61.9%) or post-and-rail fences (38.1%). In this cluster, 78.6% of pig owners reported allowing their animals to roam for food, compared to 61.7% in the cluster described above and 0% in the two other clusters in the same study area. Pigs were fed with domestic waste (83.3%) and crop by-products (47.6%). Compared to other pig farms in Arivonimamo, poultry were less often kept on the premises (64.3% vs 90.6%). Animals were less likely to receive health care (83.3%) than in the other pig farms of the region (100%), and only one third of pig owners reported visits from veterinarians or animal health workers.

#### 3.3.3 Cluster 3: farms with improved biosecurity in Arivonimamo (*n* = 38)

In these farms, pigs of exotic breeds (76.3%) or crossbred (16.7%) were kept permanently in pens made from cement (86.8%). Compared to other pig farms in Arivonimamo, more pig owners reported carrying out disinfection of their equipment (94.7% vs 13.8%). Fewer vehicles were allowed onto premises (84.2% vs 100%), and a higher proportion of farms were situated more than 100 m away from other pig farms (34.2% vs 9.2%). Animals were fed with crop by-products (86.8%) bought in shops (78.9%) rather than with domestic waste (5.3%). Commercial exchanges of pigs were made with other farmers or traders (65.8%) rather than in live animal markets (10.5%).

#### 3.3.4 Cluster 4: larger farms in Arivonimamo (*n* = 72)

This was the second largest cluster of farms in Arivonimamo. These farrow-to-finish units (73.6%) with animals from exotic breeds (95.8%) had sold more than 10 pigs in 2005 (97.2%). Animals were kept permanently in buildings with separate pens made from cement (100%). Most owners reported treating buildings against rodents (90.3%) but not disinfecting their equipment (13.9%) or treating against insects (27.8%). All farmers fed their animals with compound feeds only, which were mainly bought in markets (86.1%). Replacement animals were not bought in live animal markets but from other farmers (97.2%). In all farms, manure was collected in septic tanks. Compared to other farms in Arivonimamo, very few people working on these farms undertook other activities linked to the pig production sector (2.8% vs 78.9%).

#### 3.3.5 Cluster 5: small farms in Marovoay (*n* = 45)

For this cluster of farms, all the test-values produced *P* values higher than 0.05. It was therefore interpreted as the baseline cluster, describing the average pig farm in Marovoay area. In these small finishing (62.2%) or farrow-to-finish (37.8%) units, pigs of local breeds (42.2%) or crossbred (55.6%) were kept in pens with mud or sand floor (91.1%) and post-and-rail fences (97.8%). All farms were situated less than 100 m away from other pig farms, and 37.8% of pig owners reported exchanges of animals from different farms for natural services. In most farms, poultry (77.8%) and dogs (62.2%) were kept on premises in addition to pigs. Animals were fed with crop by-products (100%), domestic waste (82.2%) and fish, meat or blood meals (68.9%). No farmers reported carrying out disinfection or treatment against insects, and only 4.4% treated buildings against rodents.

#### 3.3.6 Cluster 6: farms with reduced person-contacts in Marovoay (*n* = 39)

In this cluster, visits from other stakeholders were not frequent: only 15.4% farmers reported visits from veterinarians and animal health workers, versus 77.5% in other farms of the area. Pig owners reported selling their animals in live animal markets (61.5%) rather than to traders or other pig farmers (35.9%). In most farms, pigs were fed domestic waste (92.3%). Feeds for pigs also included crop by-products (97.4%) and fish, meat or blood meal (46.2%) bought in markets (56.4%) and rice plants (43.6%).

#### 3.3.7 Cluster 7: farms with numerous person-contacts in Marovoay (*n* = 59)

This was the largest cluster of pig farms in Marovoay area. These farrow-to-finish units (72.9%) had herds of crossbred (66.1%) and local breeds (27.1%) pigs. Feeds were bought from rice producers and rice plants (98.3%) as well as in markets (100%). Compared to other farms in Marovoay, a high proportion of boar owners reported lending their animals for natural services (23.7% vs 3.3%). A majority of farmers reported having visits from other pig farmers (69.5%) and traders (64.4%), and allowing visitors’ vehicles on farms (69.5%). Most farmers reported selling their animals to other farmers, traders and in live animal markets (93.2%) but none reported buying replacement animals in live animal markets.

#### 3.3.8 Cluster 8: farms with improved sanitary measures in Marovoay (*n* = 38)

They were farrow-to-finish (65.8%) and finishing (31.6%) units with crossbred animals (84.2%) permanently kept in pens closed by post-and-rail fences (86.8%) or solid walls (13.2%). In 36.8% of farms in this cluster, animals were fed with compound feeds, versus 2% of farms in the rest of the area. Compared to other pig farms in the region, more farmers reported carrying out disinfection of their farm equipment (31.6% vs 2.8%) and control of rodents (57.9% vs 9.8%). Manure was used as a fertiliser for crops (36.8%) or thrown away far from premises (15.8%) rather than disposed of nearby premises as in other farms of the study area (44.7% vs 75.5%).

## 4 Discussion

This study provided a description of swine husbandry practices in 709 pig farms located in three geographic areas of Madagascar, and allowed identifying distinct groups of farms on the basis of management and biosecurity practices.

### 4.1 Method of data collection

It was not possible to select a random sample of pig farms in Madagascar, as there is no central or even village-level register of pig farms. The three study areas were a purposive sample of Malagasy regions where pig production is important. Within these areas, a multi-stage sampling approach using line transects was applied to limit selection bias. The selection of the primary sampling units, the fokontanies, could not be performed randomly due to the absence of sampling frame, the limited road network and difficult access to remote rural settlements. Therefore, the study sample may have been biased towards pig farms better connected to transport networks. Because of the non-random sampling approach applied in this study, results are relevant to the study sample but one should be cautious in making inference about the study population.

The effective sample size achieved in the study was sufficient to estimate frequencies of management and biosecurity practices at a 90% confidence level and with a precision of 5%. The data collected should thus provide acceptable estimates of the farming practices in the study sample.

Our study relied on data about farm management practices as reported by farmers, and not on direct observations. It was decided not to enter premises in order to not compromise farm biosecurity. During the pre-testing of the questionnaire, farm visits were done after completing the questionnaire, to check the validity of the responses given. It appeared that the good level of accuracy of information reported by farmers could be further improved by asking for a description of their protocols, specifically in relation to animal health care and biosecurity measures. Semi-open questions were subsequently included in the final version of the questionnaire. Positive answers were only considered validated when an appropriate description of the products and protocols used was provided for the following practices: animal health care, control of rodents and insects, cleaning and disinfection, use of proper farm clothing, use of disinfection baths or sprays at the entrance of the premises, quarantine for pigs entering the farm, presence of a sickbay, equipment shared with other farm(s), and systematic working route progressing from clean to dirty zones of the farm. Some farmers probably still gave answers reflecting what they thought they were supposed to do rather than what they actually did, and others refused to answer some questions. In order to reduce this information bias, interviewers explained the aim of the study to farmers, and emphasized that we were interested in practices actually applied on the farm, with the aim of providing adapted recommendations for limiting the risk of introduction of contagious disease into their pig herds.

Another potential source of bias in this study was the administration of questionnaires by 9 interviewers. In accordance with recommendations made by Smith and Morrow (1996) to reduce such bias, the interviewers took part in the testing of the questionnaire and their feedback was taken into account when refining questions. In addition, detailed instructions for the interviewers were provided in the questionnaire and interviewers were trained for the interview process.

### 4.2 Multivariate analyses

Multiple factor analysis and hierarchical cluster analysis are particularly well suited for this study, as they are adapted to questionnaire data with correlated variables. MFA allows the analysis of variables separated into groups according to distinct biological meanings, which enables an intuitive interpretation of the results. The use of multivariate analyses for describing management and biosecurity practices has an advantage over the scoring system used in the study by Julio Pinto and Santiago Urcelay (2003), as it can give an indication not only of the level of biosecurity in farms, but also of the main differences of practices existing between groups of farms. Other studies have used multivariate analyses to investigate swine management and biosecurity practices (Hurnik et al., 1994; Rose and Madec, 2002; Boklund et al., 2004; Ribbens et al., 2008), using either factor analysis or multiple correspondence analysis and cluster analysis. In these studies, although practices were initially presented according to different aspects of practices (e.g. management and personnel, transport to slaughter, biosecurity status), all variables were then pooled for the multivariate analysis. Only in the study by Ribbens et al. (2008) were management practices and biosecurity status considered separately, in two independent analyses. In this context, the advantage of MFA over multiple correspondence analysis is that it allows the simultaneous analysis of different aspects of husbandry and biosecurity practices.

### 4.3 Management and biosecurity practices in Malagasy smallholder pig farms

The results showed that the biosecurity is poor in Malagasy swine farms, despite a campaign implemented in 2000 to train pig owners on swine diseases – and ASF in particular – and measures to prevent them (Malagasy des Professionels de l’Elevage, unpublished data). Most pig farms were small farming systems where almost no sanitary measures were applied. In addition, many opportunities for contacts existed, such as with pigs from other farms for commercial exchanges or natural services, with other animals present on the premises and with various stakeholders within the pig production sector.

The results of the MFA showed heterogeneity between regions in terms of management and biosecurity practices. This heterogeneity may be due, partially at least, to differences in ethnicity and culture, climate and agro-systems between the three areas selected for this study.

Additional information was collected during informal interviews with veterinarians, representatives of farmer associations and other key stakeholders from the pig production sector. This information suggested that training and technical support to farmers vary between regions, and this may be another reason for the presence of distinct profiles of husbandry practices in the three regions. In both Ambatondrazaka and Arivonimamo, veterinarians and animal health workers are present in cities and work with pig farmers. A dynamic farmers’ association is also present in Arivonimamo city. In the region of Marovoay, veterinarians work mainly with zebu and beef cattle producers. A larger number of farmers reported little training and limited access to technical advice in this area, although farmers’ associations were set up in some localities. The results obtained at the regional level reflected these differences: pig farms in Marovoay had lower biosecurity standards than in Ambatondrazaka, where the pig farming systems had in turn lower biosecurity standards than in Arivonimamo.

The results of the HCA showed that within-area differences existed and different types of farms were differentiated in Arivonimamo and Marovoay areas. In Ambatondrazaka, the absence of grouping in terms of swine management and biosecurity practices suggested that pig farmers have individually adapted their practices in accordance with their own perceptions of practices influencing the introduction of contagious diseases. In Marovoay and Arivonimamo, within-area differences might be partially explained by variation in: access to professional expertise on swine health, technical support and training on farm management and diseases. Moreover, the variation observed probably reflected differences in household wealth and whether owners reared pigs for subsistence or commercial purpose. Unfortunately, this cannot be confirmed as it was not investigated by the questionnaire. Finally, in Arivonimamo and Marovoay, farmers associations aiming to improve production are present. This might be an indication of some farmers’ willingness to work together and share expertise in order to reduce disease risk and improve productivity.

The most important result of this study is the identification and characterisation of the different clusters of farms existing in the investigated study areas of Madagascar, as it will allow the development of tailored recommendations for improving productivity and reducing the risk of introduction of contagious diseases. This is essential in Madagascar, where pig owners have limited financial resources and can only make limited changes to their practices. Support to smallholder farmers should help them in making informed changes according to their management profiles and their respective influence on the risk of disease, rather than providing farmers with a long list of management practices and biosecurity measures they would never be able to all implement. Studies as the one presented here represent the first step in this direction.

In conclusion, this study provided a description of management and biosecurity practices in smallholder pig farms in three regions of Madagascar, and the identification of farm groups based on different patterns of husbandry practices. The practices investigated in this study were assumed to have a potential influence on the introduction of contagious diseases. The results presented here will be used in a subsequent study to investigate the association between the main practices implemented in Malagasy pig farms and clinical signs suggestive of ASF, CSF and Teschen disease. Results of both studies will then be used to develop tailored recommendations, in order to reduce the risk of introduction of diseases amongst the different types of smallholder pig farms identified in Madagascar.

## Conflict of interest statement

None declared.

## Figures and Tables

**Table 1:** Characteristics of the 3 study areas selected in Madagascar for the description of management and biosecurity practices in pig farms.

	Ambatondrazaka	Arivonimamo	Marovoay
Climate^a^	Wet tropical	Temperate	Wet and dry tropical
Surface (km^2^)^b^	3660	710	2150

*Herdsize*^b^			
Minimum	1	1	1
Median	3	7	4
Maximum (max. excl. piglets)	98 (98)	75 (55)	84 (45)

Ethnic group^a^	Sihanaka	Merina	Sakalava
Rice crop management^a^^,^^c^	Irrigated rainfed lowland	Rainfed lowland upland (slash-and-burn)	Irrigated
Main livestock production^d^	Zebu (agricultural work), dairy cattle, swine, small ruminants	Zebu (agricultural work), dairy cattle, swine	Zebu (meat production), dairy cattle, swine

a http://lcweb2.loc.gov/frd/cs/mgtoc.html.

b Results of the questionnaire survey conducted from December 2005 to April 2006.

c http://www.fao.org/AG/Agp/agpc/doc/riceinfo/AFRICA/Madagascar.htm.

d http://www.ilo.cornell.edu/polbrief/03conv/map3-3.html.

**Table 2:** Structure of the 709 pig farms, as reported by farmers interviewed from December 2005 to April 2006.

Variable	Frequency (%)			
	Overall (*n* = 709)	Amb^a^ (*n* = 272)	Arv^b^ (*n* = 233)	Mrv^c^ (*n* = 204)
Type of enclosure				
*Post-and-rail fence*	65.7	92.3	7.7	96.6
*Wall (mud or cement)*	34.3	7.7	92.3	3.4

Flooring				
*Mud or sand*	44.7	24.3	48.1	68.1
*Wood duckboard*	30.3	63.6	1.3	19.1
*Cement*	25.0	12.1	50.6	12.8

Roofing				
*Thatched roof*	76.3	82.7	49.8	98.0
*Tiles or metal sheet roof*	15.5	17.3	25.3	2.0
*Pen in house basement*	8.2	0.0	24.9	0.0

Number of pig pens				
*One pen*	41.6	36.4	45.5	44.1
*More than one pen*	58.4	63.6	54.5	55.9

Presence of other pig farm(s) <100 m away	90.1	93.0	86.7	90.2

a Amb: Ambatondrazaka.

b Arv: Arivonimamo.

c Mrv: Marovoay.

**Table 3:** Animal-contacts practices in the 709 Malagasy pig farms, as reported by farmers interviewed from December 2005 to April 2006.

Variable	Frequency (%)			
	Overall (*n *= 709)	Amb^a^ (*n *= 272)	Arv^b^ (*n* = 233)	Mrv^c^ (*n* = 204)
Type of confinement				
*Total*	80.8	87.1	64.4	91.2
*Partial*	19.2	12.9	35.6	8.8

Presence of poultry on the premises	83.5	84.6	85.8	79.4
Presence of dogs or cats on the premises	54.3	46.3	46.8	73.5
Presence of cattle on the premises	30.9	10.3	55.4	30.4

Origin of pigs				
*Live animal markets, other farmers*	21.7	20.2	42.5	0.0
*Other farmers only*	49.8	65.5	18.4	64.7
*Neither live animal market nor other farmers*	8.3	10.3	7.3	6.9
*Respondent did not wish to answer*	20.2	4.0	31.8	28.4

Destination of pigs				
*Live animal markets and traders, butchers, farmers*	10.9	3.3	25.7	3.9
*Traders, butchers or other farmers*	75.9	83.5	67.8	75.0
*Respondent did not wish to answer*	13.2	13.2	6.5	21.1

Boar lent to other farms for natural service	27.5	34.6	34.8	9.8
Boar from other farm used for natural service	26.5	24.6	18.0	38.7

a Amb: Ambatondrazaka.

b Arv: Arivonimamo.

c Mrv: Marovoay.

**Table 4:** Person- and vehicle-contacts in the 709 Malagasy pig farms, as reported by farmers interviewed from December 2005 to April 2006.

Variable	Frequency (%)			
	Overall (*n *= 709)	Amb^a^ (*n *= 272)	Arv^b^ (*n* = 233)	Mrv^c^ (*n* = 204)
People on the farm undertake other activities linked to the pig production sector	67.0	81.3	55.4	61.3
Visits of traders from the pig production sector	32.6	22.1	43.4	34.3
Visits of butchers selling pork meat	37.9	32.7	38.2	44.6
Visits of other pig farmers	31.2	29.8	30.9	33.3
Visits of veterinarians or animal health workers	74.9	85.3	70.0	66.7
Visits of family and friends	47.1	68.4	42.9	23.5
Vehicles allowed onto the premises	62.3	52.6	97.4	35.3

a Amb: Ambatondrazaka.

b Arv: Arivonimamo.

c Mrv: Marovoay.

**Table 5:** Feeding practices in the 709 Malagasy pig farms, as reported by farmers interviewed from December 2005 to April 2006.

Variable	Frequency (%)			
	Overall (*n *= 709)	Amb^a^ (*n *= 272)	Arv^b^ (*n* = 233)	Mrv^c^ (*n* = 204)
Pigs fed with compound feeds	17.6	8.8	33.9	10.8
Pigs fed with fish meal, blood or meat meals	40.8	49.6	0.0	75.5
Pigs fed with industrial and agricultural by-products	82.8	97.1	53.7	97.1
Pigs fed with domestic waste	59.2	51.5	48.5	81.9
Feeds bought in markets	58.4	47.8	45.5	87.2
Feeds bought in shop	13.8	0.7	27.0	16.2
Feeds bought from rice plants	43.3	61.8	2.6	65.2

a Amb: Ambatondrazaka.

b Arv: Arivonimamo.

c Mrv: Marovoay.

**Table 6:** Sanitary practices in the 709 Malagasy pig farms, as reported by farmers interviewed from December 2005 to April 2006.

Variable	Frequency (%)			
	Overall (*n *= 709)	Amb^a^ (*n *= 272)	Arv^b^ (*n* = 233)	Mrv^c^ (*n* = 204)
Health care provided to pigs	91.0	98.2	97.0	74.5
Re-usable syringes kept on farm for care to pigs	31.3	22.8	53.6	17.2
Insecticide treatment on premises	11.9	16.2	14.2	3.4
Treatment against rodents on premises	36.8	36.4	53.2	18.6
Disinfection of equipment on premises	17.4	15.4	27.0	8.8

Management of manure				
*Collected in a septic tank*	15.8	8.1	37.8	1.0
*Used as a fertiliser for crops*	41.5	50.4	56.7	12.3
*Discarded nearby to premises*	27.6	20.2	0.4	68.6
*Thrown away far from premises or sold*	15.1	21.3	5.1	18.1

Slaughtering of pigs on the premises (occasional or regular)	17.9	8.5	24.9	22.6

a Amb: Ambatondrazaka.

b Arv: Arivonimamo.

c Mrv: Marovoay.

**Table 7:** Demographics (Supplementary variables) in the 709 Malagasy pig farms, as reported by interviewed from December 2005 to April 2006.

Variable	Frequency (%)			
	Overall (*n *= 709)	Amb^a^ (*n *= 272)	Arv^b^ (*n* = 233)	Mrv^c^ (*n* = 204)
Type of farm				
*Breeding unit*	7.0	9.2	9.4	1.0
*Farrow-to-finish unit*	40.2	29.0	40.8	54.9
*Finishing unit*	52.8	61.8	49.8	44.1

Breed(s) of pigs				
*Exotic breed*	33.5	45.2	43.8	6.4
*Local breed*	30.8	17.7	50.6	25.5
*Crossbred animals*	35.7	37.1	5.6	68.1

Presence of a boar	11.4	8.5	10.3	16.7

Number of sows				
*0*	53.9	64.0	49.8	45.1
*1–2*	39.2	33.1	38.6	48.0
*>2*	6.9	2.9	11.6	6.9

Number of finishing pigs				
*0*	16.8	18.0	15.0	17.1
*1–5*	66.3	75.7	55.4	66.2
*>5*	16.9	6.3	29.6	16.7

Number of unweaned piglets				
*0*	70.8	78.7	60.5	72.1
*1–10*	22.6	20.2	23.6	24.5
*>10*	6.6	1.1	15.9	3.4

Number of pigs sold in 2005				
*1–10*	82.6	94.1	55.8	98.0
*>10*	17.4	5.9	44.2	2.0

a Amb: Ambatondrazaka.

b Arv: Arivonimamo.

c Mrv: Marovoay.

**Table 8:** Management and biosecurity practices in the 8 clusters identified with the hierarchical cluster analysis: 4 in Arivonimamo (233 farms) and 4 in Marovoay (181 farms).

Variable	Arivonimamo				Marovoay			
	Cluster 1 (*n* = 81)	Cluster 2 (*n* = 42)	Cluster 3 (*n* = 38)	Cluster 4 (*n* = 72)	Cluster 5 (*n* = 45)	Cluster 6 (*n* = 39)	Cluster 7 (*n* = 59)	Cluster 8 (*n* = 38)
Type of farm								
*Breeding unit*	10	10*	0	2	0	1	0	1
*Farrow-to-finish unit*	13	6	23	53*	17	15	43	25
*Finishing unit*	58	26	15	17	28	23	16	12

Breed(s) of pigs								
*Exotic breed*	3*	1*	29	69*	1	2	4	3
*Crossbred animals*	3	0	7*	3	25	27	39	32
*Local breed*	75*	41*	2*	0*	19	10	16	3

Number of finishing pigs								
*0*	13	10	0	12	9	12	9	3
*1–5*	68	29	18	14*	34	25	31	27
*>5*	0*	3	20	46*	2	2	19	8
Less than 10 pigs sold in 2005	77*	31	20	2*	45	39	57	37

Enclosure: Solid wall (and not post-and-rail)	81	26*	37	71	1	0	1	5*

Flooring								
*Mud or Sand*	70*	39*	3	0*	4	35	27	20
*Cement*	10*	3*	33	72*	3	2	11	8
*Wood duckboard*	1	0	2*	0	1	2	21	10

Roofing								
*Thatched roof*	34	27	12	43	45	39	58	37
*Tiles or Metal sheet roof*	4	0	26*	29	0	0	1	1
*Pen in basement*	43*	15	0	0	0	0	0	0

More than one pig pen	19	13	25	70	17	8	45	30
Presence of other pig farms <100 m away	78	29	25*	70	45	36	54	31
Partial confinement	50	33	0	0*	5	9	3	1
Presence of poultry on the premises	77	27*	29	67	35	24	54	33
Presence of dogs or cats on the premises	15	19	11	64*	28	18	52	35
Presence of cattle on the premises	74*	33	8	14	14	10	21	10

Origin of pigs								
*Live animal markets and other farmers*	71*	23	4	1*	0	0	0	0
*Other farmers only*	8	14	25*	70	45	36	58	28
*Neither live animal market nor other farmers*	2	5	9*	1	0	3	1	10

Destination of pigs								
*Live animal markets and traders, butchers or other farmers*	35	24	5	5	6	24*	2	7
*Traders, butchers, other farmers*	41	18	32	67	39	14*	55	27
*None of the above*	5	0	1	0	0	1	2	4

Boar lent to other farms for natural service	11	15	21	41	0	0	14*	4
Boar from other farm used for natural service	16	6	15	8	17	14	26	15
People on farm undertake other activities linked to the pig production sector	76*	23	28	2*	21	17	46	35
Visits of traders	36	9	9	47	18	1	38*	5
Visits of other pig farmers	15	24	14	19	16	1	41*	7
Visits of veterinarians or animal health workers	55	14*	26	68	38	6*	55	17
Vehicles allowed onto the premises	81	42	32*	72	16	0	41*	5
Pigs fed with compound feeds	1	0	6	72*	0	2	1	14*
Pigs fed with fish meal, blood or meat meals	0	0	0	0	31	18*	57	33
Pigs fed with industrial and agricultural by-products	72*	20	33	0*	45	38	59	35*
Pigs fed with domestic waste	76*	35	2*	0*	37	36	50	26
Feeds bought in markets	37	2*	5	62*	43	22*	59	35
Feeds bought in shop	28	5	30*	0*	0	11	0	19*
Feeds bought from rice plants	6	0	0	0	40	17	58*	2*
Health care provided to pigs	81	35*	38	72	35	23	48	29
Control of rodents	30	17	12	65*	2	11	1	22*
Control of insects	2	1	10	20	0	3	1	2
Disinfection of equipment on premises	4	13	36*	10	0	3	1	12*

Management of manure								
*Collected in septic tank*	3*	12	1	72*	0	0	0	1
*Thrown away far from premises or sold*	0	2	10*	0	5	1	20	6
*Used as a fertiliser for crops*	78*	27	27	0*	2	3	4	14*
*Discarded nearby to premises*	0	1	0	0	38	35	35	17

Results are count of positive answers on variables of the questionnaire administered from December 2005 to April 2006.

* *P* < 0.05 for the test value. It indicates that in the given cluster, the proportion of positive answers for this variable category is different from the proportion in all pig farms of the study area.
